# Supplementary material for: Systematic Analysis of Driving Modes and NiFe Layer Thickness in Planar Hall Magnetoresistance Sensors
Source: Sensors (Basel). 2025 Feb 18;25(4):1235. doi: 10.3390/s25041235 (PMC11860601; doi:10.3390/s25041235)
Supplement: Supplementary file 1 [file sensors-25-01235-s001.zip › sensors-3440362-supplementary.pdf]

Systematic Analysis of Driving Modes and NiFe Layer Thickness in Planar Hall Magnetoresistance Sensors

Changyeop Jeon[a,1], Mijin Kim[a], Jinwoo Kim[a], Sunghee Yang[a], Eunseo Choi[a], and Byeonghwa Lim[b]*

[a]*Department of Physics and Chemistry, DGIST, Daegu, 42988, South Korea*
[b]*Department of Smart Sensor Engineering, Andong National University, Gyeongsangbyk-do, 36729, South Korea*

*Corresponding authors. E-mail addresses: *Limbh@anu.ac.kr* (Byeonghwa Lim).

1. Resistivity Variation According to NiFe thickness

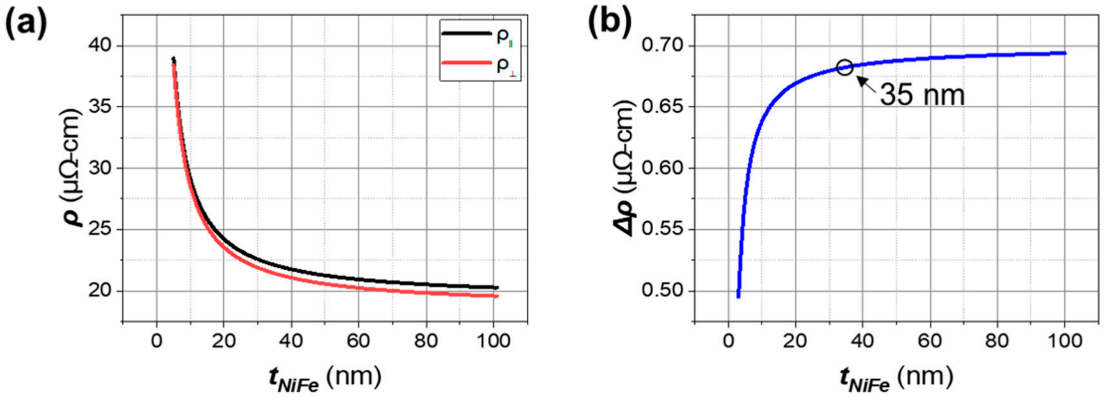

Figure S1. Resistivity and $\Delta\rho$ characteristics as a function of FM layer thickness. (a) Resistivity ($\rho_{//}$ and $\rho_{\perp}$) as a function of NiFe thickness, showing the decrease in resistivity with increasing thickness. (b) Relative change in $\Delta\rho$ as a function of NiFe Thickness.

According to the Fuchs-Sondheimer theory, the resistivity of a thin metallic film, like NiFe, depends on its thickness due to surface scattering effects. As the thickness of the NiFe layer increases, the influence of surface scattering diminishes, and the resistivity gradually approaches the bulk resistivity value.

2. Noise Spectrum Curve of the Cross-Type PHMR Sensor

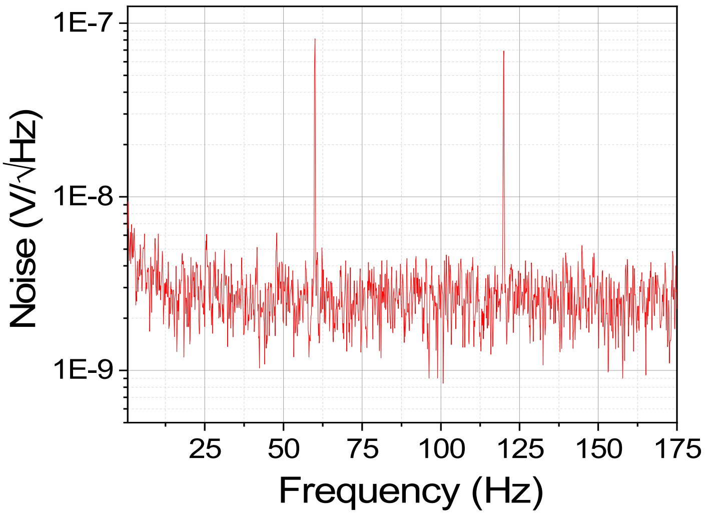

Figure S3. Noise spectrum of the cross-type PHMR sensor measured using Spectrum Analyzer.

This figure illustrates the noise spectrum of the cross-type PHMR sensor. The noise level is approximately 2.4 nV/√Hz in the frequency range of interest, indicating the sensor's low intrinsic noise characteristics.

3. Sensor Output Characteristics for Connected Load Resistance as a Function of Drive Mode and NiFe Layer Thickness.

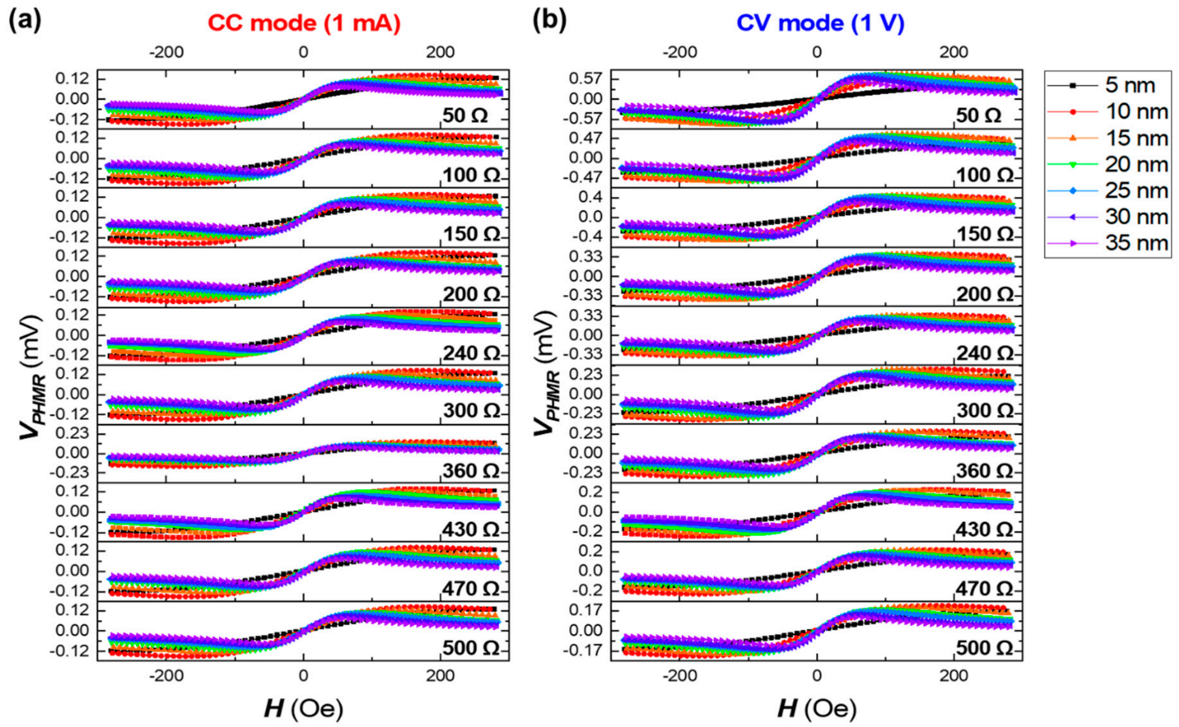

Figure S2**.** Comparison of PHMR sensor signal response based on NiFe layer thickness and load resistance. Magnetic field response of PHMR sensor in CC mode (a) and CV mode (b) across various NiFe layer thicknesses ranging from 5 nm to 35 nm and Load resistance ranging from 50 Ω to 500 Ω.

The graph displays the output characteristics of a sensor for various NiFe layer thicknesses (5 nm to 35 nm) under two different drive modes: constant current (CC mode, 1 mA) on the left and constant voltage (CV mode, 1 V) on the right. Each line represents the sensor output for a specific NiFe thickness, with distinct colors indicating different thickness values. In both CC and CV modes, the sensor output varies depending on the thickness of the NiFe layer, affecting the load resistance response.
